# GEMIN6 Overexpression Correlates with the Low Immune Cell Infiltration and Poor Prognosis in Lung Adenocarcinoma

**DOI:** 10.1155/2022/1930604

**Published:** 2022-10-15

**Authors:** Yunsong Peng, Zeng Wang, Jianyong Cai, Xiaoqiao Dong, Quan Du

**Affiliations:** ^1^Department of Pharmacy, The Cancer Hospital of the University of Chinese Academy of Sciences (Zhejiang Cancer Hospital), Institute of Basic Medicine and Cancer (IBMC), Chinese Academy of Sciences, Hangzhou, 310022 Zhejiang, China; ^2^Department of Neurosurgery, The Wenzhou Central Hospital, Wenzhou, 325000 Zhejiang, China; ^3^Department of Neurosurgery, Affiliated Hangzhou First People's Hospital, Zhejiang University School of Medicine, Hangzhou, 310006 Zhejiang, China

## Abstract

**Background:**

Gem nuclear organelle-associated protein 6 (GEMIN6) is a component of the GEMINS protein family involved in the survival of motor neuron (SMN) complex. SMN interfered with snRNP assembly and mRNA processing resulting in tumorigenesis. We performed this study to explore the association between GEMIN6 and lung adenocarcinoma (LUAD).

**Methods:**

We used The Cancer Genome Atlas (TCGA) and Gene Expression Omnibus (GEO) databases to collect transcriptomic expression data of LUAD patients and analyze the difference in GEMIN6 expression between normal and tumor tissues of LUAD. qRT-PCR analysis was also performed to detect the expression of GEMIN6 in normal and LUAD cells. The expression of GEMIN6 on the LUAD patient survival outcome was estimated by the Kaplan–Meier curves and Cox analyses. In addition, the Metascape online tool and single-sample GSEA were employed to find out the underlying biological mechanisms of GEMIN6. Furthermore, the correlations of GEMIN6 expression with immune cell infiltration in LUAD were analyzed by ssGSEA and Spearman correlation analysis.

**Results:**

Compared with the normal tissues and cells, the expression of GEMIN6 was significantly higher in LUAD tissues and cells; the high expression GEMIN6 was also found in the advanced pathologic stage and advanced N and T stages of LUAD. GEMIN6 high expression was significantly associated with inferior overall survival. The heat map revealed the top 20 coexpressed genes with GEMIN6, including SF3B6, CPSF3, and PSMB3. Functional enrichment analysis demonstrated that enrichment genes are associated with the cell cycle, mRNA processing, and energy metabolism. Additionally, GEMIN6 was negatively related to the immune cell infiltration in LUAD.

**Conclusions:**

This study demonstrated that GEMIN6 was involved in the tumorigenesis and progression of LUAD. GEMIN6 could be an important molecular marker of poor prognosis and a therapeutic target of LUAD.

## 1. Introduction

Lung cancer, including lung adenocarcinoma (LUAD) and lung squamous cell carcinoma, is the principal cause of cancer-related death and the second most commonly occurring cancer worldwide, accounting for 18.0% of deaths and 11.4% of cancers diagnosed [1]. The prolongation in survival has been steadily increased for LUAD due to improvements in treatment options in recent years, including molecular-targeted therapy and immunotherapy. Despite these developments, however, the 5-year survival rate is merely 21%, which is remarkably lower than other common cancers [2]. Therefore, determining new molecular pathways and efficient targets is urgent for patients with LUAD.

MicroRNAs are single-stranded noncoding RNAs that play a critical role in silencing target mRNAs. They can aberrantly express in human cancers and be involved in carcinogenesis and cancer progression [3, 4]. Gem nuclear organelle-associated protein 6 (GEMIN6), a component of the GEMINS protein family, can oligomerize and form the survival of motor neuron (SMN) complex. SMN interfere with a number of cellular RNA metabolisms comprising small nuclear ribonucleoprotein (snRNPs) assembly [5–7]. Several prior studies found the association between GEMIN4 and cancer progression, including bladder cancer [8], renal cancer [9], lung cancer [10], and prostate cancer [11] that yield a preliminary insight that the GEMINS protein family was related to the development of malignant tumors. By contrast, studies focused on GEMIN6 are rare, especially on cancer research. In nonsmall cell lung cancer, GEMIN6 was found to accelerate AURKB maturation and c-Myc stabilisation to promote the cancer progression [12], while the clear role of GEMIN6 in LUAD remains to be addressed.

Given that, we supposed that GEMIN6 might be involved in LUAD development and could be a potential therapeutic target for LUAD. Herein, we performed this study to explore the role and functions of GEMIN6 in LUAD. The expression levels and impact of GEMIN6 in LUAD were estimated by analyzing data from The Cancer Genome Atlas (TCGA) database. An independent dataset, named the Gene Expression Omnibus (GEO) database, was employed to verify the impact of GEMIN6. The relationship between GEMIN6 expression and clinical characteristics was analyzed by Cox analyses. Additionally, the Kyoto Encyclopedia of Genes and Genomes (KEGG) and Gene Ontology (GO) analyses were constructed to explore the potential biological mechanisms of GEMIN6. The correlation between GEMIN6 and the immune microenvironment was identified by the single-sample gene set enrichment analysis (ssGSEA).

## 2. Materials and Methods

### 2.1. Data Sources from TCGA and GEO

Firstly, the GEMIN6 expression in pan-cancer was analyzed by the TIMER web-interactive tool [13]. We downloaded the RNA-seq data (FKPM level) and clinicopathological data of LUAD patients from the TGCA database. The patient samples without survival information were excluded. A total of 535 samples were screened for the next step of research. Moreover, in order to eliminate the technical error of the RNA sequencing data, we standardized the data by R software (V3.6.2). FPKM level data was transformed into the TPM (transcripts per million reads) level for the following analysis. The GSE31210 database was downloaded from NCBI as a verification dataset. The detailed clinicopathological features of all samples were shown in Table 1.

### 2.2. Coexpressed Gene and Functional Enrichment Analysis

R software was used to screen the genes coexpressed with GEMIN6. Spearman correlation analysis was used to examine the correlation between GEMIN6 and coexpressed genes. The top 20 coexpressed genes were shown by the heat map. Metascape (https://metascape.org), an excellent online analysis tool, has the function of functional enrichment and related pathway analyses [14], which was employed for functional enrichment of GEMIN6 coexpressed genes. Statistical significance was identified as *p* value < 0.05, with a minimum gene count = 3 and an enrichment factor > 1.0. Moreover, to further explore the GEMIN6-related signaling pathway, GSEA analysis was carried out using the clusterProfiler package [15]. The following conditions are considered to be statistically significant: |NES| > 3 and *p* value < 0.001.

### 2.3. Prognostic Model

Based on the significant clinical variables from multivariate Cox regression analysis, we further constructed a nomogram as a model for predicting the prognosis of patients with LUAD using R package rms. In accordance with the formula multivariate Cox regression model, the risk score (RS) of each sample was evaluated. Afterward, the samples were categorized as low-risk groups and high-risk groups according to the median value of RS.

### 2.4. Analysis of Immune Infiltration and GEMIN6 Expression by ssGSEA

To explore the effect of GEMIN6 expression on the immune microenvironment, ssGSEA was performed by the GSVA Package in R [16]. We calculated 24 types of immune cell infiltration according to the expression of immune-related genes from the published gene signature list [17]. Furthermore, the Spearman correlation was carried out to evaluate the relationship of the different immune cell infiltration and GEMIN6 expression.

### 2.5. Cell Culture

The A549, H1299, SK-MES-1, PC-9, and NCI-H23 LUAD cell lines and the normal human bronchial epithelial cell line BEAS-2B were purchased from the iCell Bioscience Inc. (China). A549, H1299, SK-MES-1, PC-9, and NCI-H23 were cultured in RPMI-1640 (iCell Bioscience Inc., Shanghai, China) supplemented with 10% FBS at 37°C containing 5% CO_2_; BEAS-2B cells were cultured in DMEM (iCell Bioscience Inc., Shanghai, China) and 10% FBS at 37°C containing 5% CO_2_.

### 2.6. qRT-PCR Analysis

Total RNAs were extracted from cells with MolPure® TRIeasy Plus Total RNA Kit (Yeasen Biotechnology (Shanghai) Co. Ltd., China) and were reversed transcribed into cDNA using the Reverse Transcription Kit (CoWin Bioscience, China). For RT-PCR, the cDNA was amplified using SYBR Premix Ex Taq (Yeasen Biotechnology (Shanghai) Co. Ltd., China) and run on LightCycler® 96 (Roche, Germany). Relative mRNA expression was counted using the 2^−ΔΔ*CT*^ method. The primer sequences of GEMIN6 and *β*-actin were listed: GEMIN6 forward: 5′-ATTTACAAAGAGGTCCGAGTGAC-3′, reverse 5′-AGCATGTCCCATAATTCCGGT-3′; *β*-actin forward: 5′-CATGTACGTTGCTATCCAGGC-3′, reverse 5′-CTCCTTAATGTCACGCACGAT-3′.

### 2.7. Statistical Analysis

The Wilcoxon rank-sum test was applied to estimate the discrepancy between GEMIN6 expression levels in LUAD tissue and normal tissue. The association between clinical factors and the expression level of GEMIN6 was evaluated with the Wilcoxon single-rank test and Kruskal-Wallis test. With the log-rank test, Kaplan-Meier curves were utilized to evaluate the statistical differences in OS between these two different expression groups. Moreover, multivariate analyses based on the Cox regression model were applied to assess the prognosis. Differences in measurement data among more than two groups were analyzed using ANOVA with the post-Tukey test. Only *p* values < 0.05 were considered statistically significant.

## 3. Results

### 3.1. Study Characteristics

A total of 535 unique LUAD individuals were collected based on TCGA database. The following parameters, incorporating demographic and clinicopathological ones were retrieved, including age, gender, race, smoking habit, TNM stages, pathological stages, and GEMIN6 expression. The detailed characteristics of eligible cases was presented in Table 1.

### 3.2. Expression Status of GEMIN6 in LUAD Tissues

**Figure 1:**
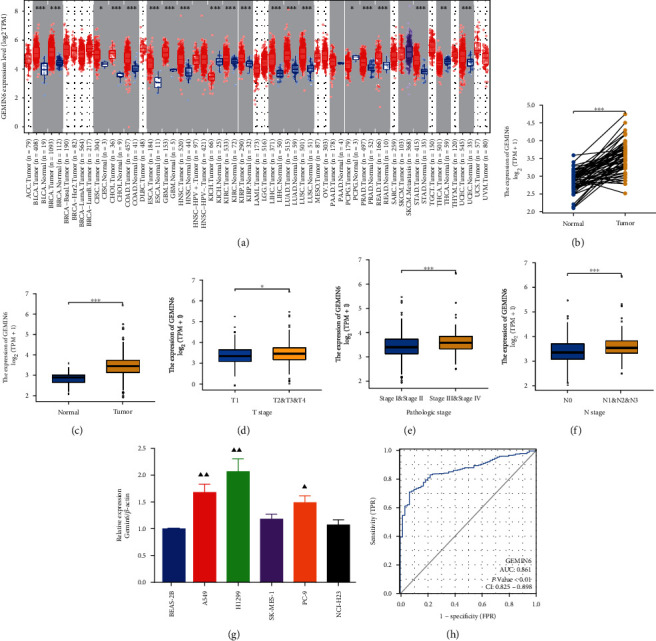
GEMIN6 expression in LUAD and other types of human cancers from TCGA data. (a) GEMIN6 expression in different tumor types; (b) the expression of GEMIN6 in LUAD and its paired adjacent tissues; (c) the expression of GEMIN6 in LUAD and normal tissues; (d) the association of GEMIN6 expression and T stages in LUAD; (e) the association of GEMIN6 expression and pathologic stages in LUAD; (f) the association of GEMIN6 expression and N stages in LUAD; (g) relative expression of GEMIN6 in human bronchial epithelial cells (BEAS-2B) and LUAD cell lines (A549, H1299, SK-MES-1, PC-9, and NCI-H23); (h) receiver operating characteristic analysis (ROC) of GEMIN6 in LUAD.

Compared with the normal tissue, GEMIN6 was expressed remarkably higher in a variety of tumor tissues, including LUAD (Figure 1(a)). Compared with the normal tissue, GEMIN6 was expressed remarkably higher in LUAD tissues based on the Wilcoxon rank-sum test (*p* < 0.001) (Figures 1(b) and 1(c)). Notably, compared to the low-GEMIN6 expression group, the high-expression group presented with a higher percentage of the advanced pathologic stage (*p* < 0.001), advanced N stage (*p* < 0.001), and advanced T stage (*p* < 0.05) (Figures 1(d)–1(f)). We also performed cell-level experiment, to detect the expression of GEMIN6 in normal and LUAD cells; the result showed that GEMIN6 was highly expressed in LUAD cell lines (A549, H1299, SK-MES-1, PC-9, and NCI-H23) compared to the normal human bronchial epithelial cells (BEAS-2B) (Figure 1(g)). Additionally, ROC analysis of GEMIN6 in LUAD showed that the AUC value was 0.861 (CI: 0.825−0.898, *p* < 0.01) (Figure 1(h)).

### 3.3. The Impact of GEMIN6 on the Survival of LUAD Patients

**Figure 2:**
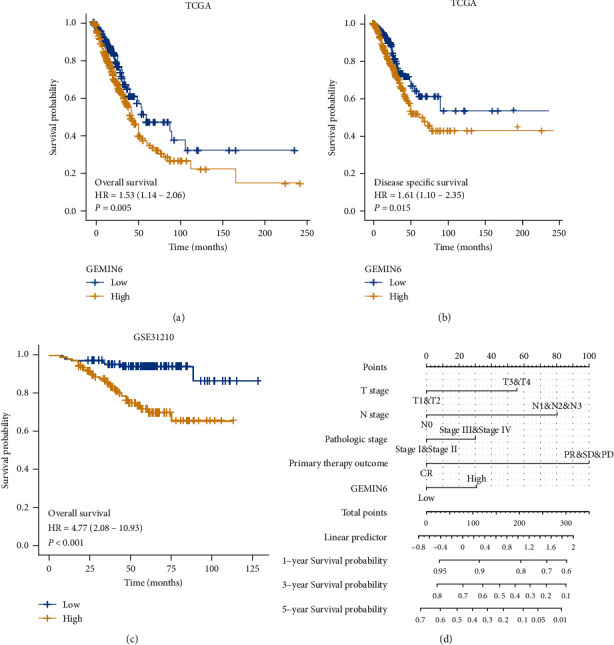
The prognostic value of GEMIN6 expression in LUAD. (a) Kaplan-Meier curves of OS based on the high or low GEMIN6 expression in the TCGA cohort; (b) Kaplan-Meier curves of DSS based on the high or low GEMIN6 expression in the TCGA cohort; (c) Kaplan-Meier curves of OS based on the high or low GEMIN6 expression in the GSE31210 cohort; (d) a nomogram that integrates GEMIN6 and other prognostic factors in LUAD from TCGA data.

The effect of GEMIN6 on the prognosis of LUAD patients was also assessed. According to Kaplan–Meier analysis, LUAD patients with GEMIN6 high expression were remarkably associated with a poor overall survival (OS) than those with GEMIN6 low expression (log-rank tests, *p* = 0.005) (Figure 2(a)). Also, the same tendency was observed in the disease-specific survival (DSS) of these two groups (*p* = 0.015) (Figure 2(b)). The GSE31210 dataset, originating from the GEO database, was employed to further verify the relationship between OS and GEMIN6 expression levels. Similarly, LUAD patients with GEMIN6 high expression were markedly related to an inferior OS to those with GEMIN6 low expression (*p* < 0.001) (Figure 2(c)). A nomogram integrating GEMIN6 and other factors that affect the prognosis of LUAD from TCGA data was presented in Figure 2(d).

In addition, the univariate and multivariate analyses of overall survival according to GEMIN6 expression and other prognostic factors were also performed; the comprehensive information was listed in Table 2. Compared to the GEMIN6 low-expression group, the univariate Cox analysis indicated that the GEMIN6 high-expression group had a poorer OS (hazard ratio (HR): 1.529, 95% CI: 1.136-2.058, *p* = 0.005). In addition, the following factors were related to a worse OS in LUAD patients: advanced T stage (HR: 2.317, 95% CI: 1.591-3.375, *p* < 0.001), advanced N stage (HR: 2.601, 95% CI: 1.944-3.480, *p* < 0.001), advanced pathologic stage (HR: 2.664, 95% CI: 1.960-3.621, *p* < 0.001), and unfavorable response group (HR: 2.690, 95% CI: 1.918-3.771, *p* < 0.001). However, there was no obvious difference in the gender, race, age, smoker, or pack-year sets.

Compared to the GEMIN6 low expression group, the multivariate Cox analysis implied that the GEMIN6 high expression group had a poorer OS following adjusting of the pathologic stage, primary therapy outcome, T stage, and N stage (HR: 1.491, 95% CI: 1.063-2.092, *p* = 0.021). The detailed results were presented in Table 2. Additionally, primary therapy outcome, T stage, and N stage were risk factors for the OS of LUAD patients.

A nomogram, integrating GEMIN6 expression, pathologic stage, primary therapy outcome, T stage, and N stage, was built (Figure 2(d)) according to multivariate Cox analysis and the needs of clinical practice. Total points could be obtained from this nomogram, and the higher total point on the nomogram indicated an inferior prognosis.

### 3.4. GEMIN6-Related Functional Enrichment Analysis

**Figure 3:**
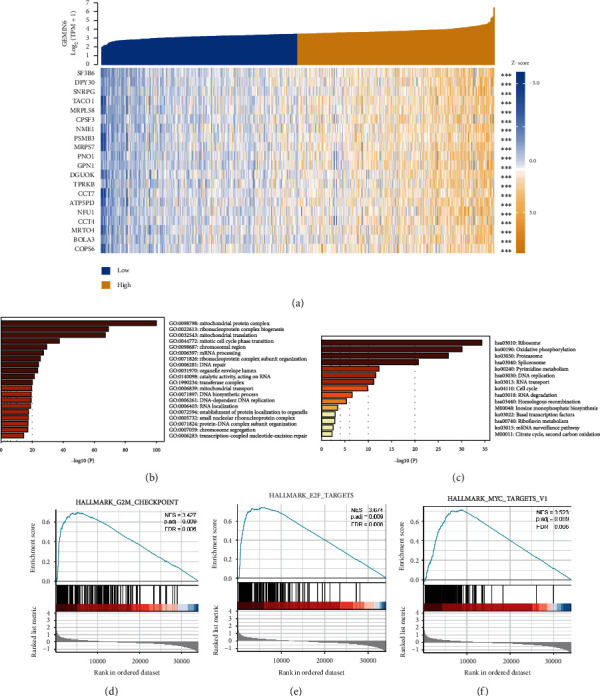
Functional enrichment analysis of GEMIN6 in LUAD. (a) Heatmap of the top 20 coexpressed genes related to GEMIN6; (b, c) GO and KEGG enrichment analysis of GEMIN6-related genes; (d, f) enrichment of genes involved in the G2M checkpoint, E2F targets, and MYC targets, as revealed by GSEA.

Based on the significant role of GEMIN6 in LUAD, the genes coexpressed with GEMIN6 were also identified. The heat map revealed the top 20 coexpressed genes with GEMIN6, including SF3B6, CPSF3, and PSMB3 (Figure 3(a)). The GO enrichment analysis of genes demonstrated several GEMIN6-related terms in three kinds of functional groups (Figure 3(b)). In the group of cellular components, GEMIN6 was largely involved in the mitochondrial protein complex, ribonucleoprotein complex biogenesis, cell cycle, mRNA processing, and DNA repair. The KEGG pathway revealed that GEMIN6 was largely involved in the ribosome, oxidative phosphorylation, spliceosome, DNA replication, and cell cycle (Figure 3(c)). Furthermore, GSEA analysis was utilized to explore the potential signaling pathways between the low- and high-GEMIN6 expression groups, based on the dataset from MSigDB Collection (h.all.v7.2.symbols.gmt). Our results revealed that the G2M checkpoint, MYC target, and E2F target signaling pathways were highly enriched in patients with overexpression of GEMIN6 (Figures 3(d)–3(f)).

### 3.5. The Correlations between GEMIN6 Expression and Immune Cell Infiltration

**Figure 4:**
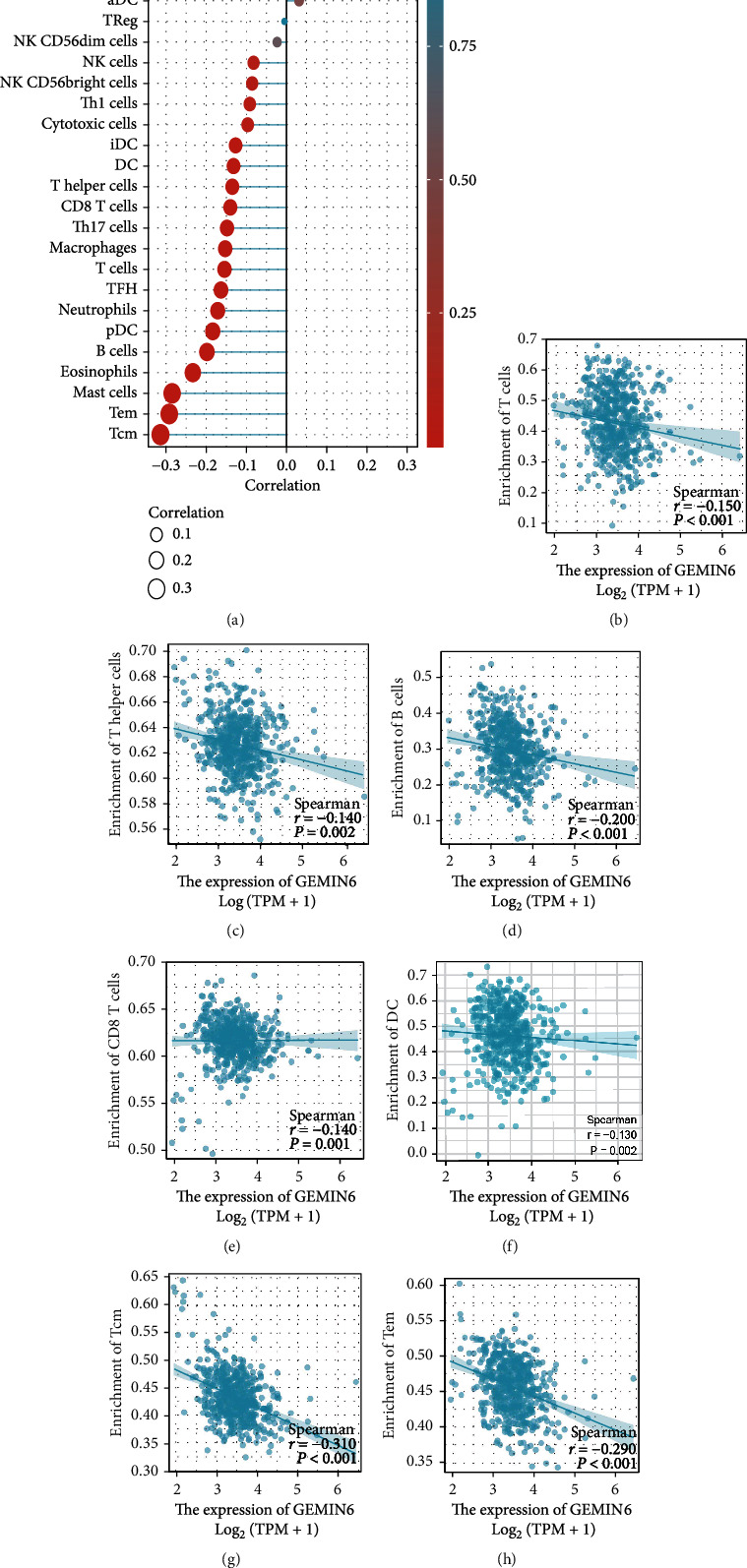
ssGSEA analyses of GEMIN6 and the correlation of GEMIN6 expression with immune cell infiltration in LUAD. (a) The correlation between the infiltration of immune cells and the expression of GEMIN6; GEMIN6 expression negatively correlates with infiltration of (b) T cells, (c) T helper cells, (d) B cells, (e) CD8+ T cells, (f) DC, (g) Tcm, and (h) Tem.

According to the lollipop chart, type 2 T helper cells (Th2) were positively correlated with the level of GEMIN6 expression. Nevertheless, it is worth noting that the expression level of GEMIN6 was negatively associated with T cells, effector memory T cell (Tem), T helper cells, B cells, CD8+ T cell, dendritic cell, and central memory T cell. The detailed results were presented in Figures 4(a)–4(h).

## 4. Discussion

Despite that promising progress has been made for LUAO therapy over the last decades, the 5-year OS remains merely 21% which was significantly lower than other common cancers [2]. Currently, chemotherapy and targeted therapy are the major treatment strategies for advanced LUAD. However, drug resistance remains the main obstacle for enhancing the clinical outcome of patients with LUAD [18]. Hence, exploring novel molecular mechanisms and efficient therapeutic targets is crucial to improving the OS of patients with LUAD.

This study showed that the expression of GEMIN6 was significantly higher in tumor tissues than normal tissues, particularly in LUAD, which implied that GEMIN6 might be involved in lung carcinogenesis. GEMIN6 high expression was related to an inferior outcome compared with GEMIN6 low expression, indicating that GEMIN6 could be considered as a promising prognostic biomarker in LUAD. Several studies documented that GEMIN4 facilitated cancer cell proliferation in renal cell carcinoma and lung cancer [10, 19]. Verma et al. [19] proposed that genetic alteration of miRNAs was related to cancer development and progression. GEMIN4 regulated snRNP assembly and mRNA processing via the SMN complex [5–7]. Based on those findings, GEMIN6, as a member of the GEMINS protein family, might also impact LUAD with a similar mechanism.

Growing evidence suggests that the GEMINS protein family works as an oncogene and is associated with cancer progression [11, 12, 20]. This study demonstrated that GEMIN6 was remarkably expressed in the advanced T stage, the advanced N stage, and the advanced pathologic stage LUAD, suggesting that GEMIN6 was potentially related to high malignant biological behavior of LUAD. The results of our study provided a basis for clinicians to evaluate and identify high-risk LUAD populations with highly malignant biological behavior.

The heat map in this study also revealed the top 20 GEMIN6-coexpressed genes, including SF3B6, CPSF3, and PSMB3. Tumorigenesis triggered by GEMIN6 might be attributed to suppressing p53 activity via SF3B6 [21], silencing spliceosome Sm gene expression through PSMB3 [22], and DNA hypermethylation by CPSF3 [23]. Based on these evidence, GEMIN6 was considered as an oncogene and therapeutic target of LUAD. GEMIN6 was a subunit of the SMN complex, which played an important role in the assembly of the spliceosomal snRNPs [5]. However, few reports have revealed the role of GEMIN6 in tumors. The results of functional enrichment analysis indicated that GEMIN6 was highly correlated with the biogenesis and assembly of snRNP, which was consistent with the previous reports. Meanwhile, we also found that GEMIN6 was involved in cell proliferation, including the cell cycle and DNA replication. Considering the worse prognosis of patients with overexpression of GEMIN6, we speculated that the high GEMIN6 expression could promote the progression of cancer via participating in cell cycle and replication in LUAD.

With the advent of immunotherapy, the tumor microenvironment is a hot topic in the current research. Previous studies have revealed that NSCLC patients with high T lymphocyte infiltration such as CD8+ T cells and CD4+ T cells were related to better OS and effective immunotherapy, compared with patients with low immune cell infiltration [24–26]. Some published studies also reveal the clues of GEMIN genes and immune cells. In the absence of regulatory T cells in scurfy mice, the myopathy-specific autoantibody profile revealed significantly increased the levels of anti-SMN as well as anti-Gemin3 antibodies in scurfy sera [27]. Gao et al. identified GEMIN3 (rs197412) which was independently associated with overall survival in non-Hodgkin's lymphoma patients, and the prognostic value of GEMIN3 in patient outcomes was also observed in the diffuse large B-cell lymphoma and T-cell lymphoma non-Hodgkin's lymphoma subtypes [28]. Importantly, our results indicated that GEMIN6 expression levels were negatively associated with immune cell infiltration, including T helper cells, CD8+ T cells, B cells, dendritic cells, and memory T cells. Although the *R* values were not so high, the *p* values were all obviously less than 0.05. We have to admitted that the results of correlation analysis were not so satisfactory, but these results do indicate that the GEMIN6 expression level might impact the ecology of the immune microenvironment, resulting in a worse prognosis of patients with LUAD. Further analysis is needed in our following study to clear the correlations between GEMIN6 expression and immune cell infiltration in LUAD.

Despite that our findings yield insights into the association between GEMIN6 and LUAD, there were several limitations in this study. Firstly, the data were originated from public databases with unknown quality control. Further studies should be performed to validate the results. Additionally, given the absence of detailed data, we cannot evaluate the role of clinical factors related to LUAD comprehensively. Besides, cellular and clinical experiments should be carried out to elucidate the association between GEMIN6 expression at the mRNA and protein levels. Finally, we offered the underlying mechanisms for GEMIN6 in LUAD; the future research direction should focus on revealing direct mechanisms.

## 5. Conclusions

In conclusion, this study provided a comprehensive insight that GEMIN6 was involved in the tumorigenesis and progression of LUAD. Our findings implied that GEMIN6 could be an important molecular marker of poor prognosis and an underlying therapeutic target of LUAD. Additionally, GEMIN6 could be a predictive biomarker of LUAD patients with immunotherapy.

## Figures and Tables

**Table 1:** Clinical characteristics of the enrolled LUAD cases from the TCGA database.

Characteristic	Levels	Overall
*n*		535

T stage, *n* (%)	T1	175 (32.9%)
	T2	289 (54.3%)
	T3	49 (9.2%)
	T4	19 (3.6%)

N stage, *n* (%)	N0	348 (67.1%)
	N1	95 (18.3%)
	N2	74 (14.3%)
	N3	2 (0.4%)

M stage, *n* (%)	M0	361 (93.5%)
	M1	25 (6.5%)

Pathologic stage, *n* (%)	Stage I	294 (55.8%)
	Stage II	123 (23.3%)
	Stage III	84 (15.9%)
	Stage IV	26 (4.9%)

Gender, *n* (%)	Female	286 (53.5%)
	Male	249 (46.5%)

Race, *n* (%)	Asian	7 (1.5%)
	Black or African American	55 (11.8%)
	White	406 (86.8%)

Age, *n* (%)	≤65	255 (49.4%)
	>65	261 (50.6%)

Smoker, *n* (%)	No	75 (14.4%)
	Yes	446 (85.6%)

Number pack years smoked, *n* (%)	<40	188 (50.9%)
	≥40	181 (49.1%)

GEMIN6 expression	Low	267 (49.9%)
	High	268 (50.1%)

**Table 2:** The univariate and multivariate analyses of overall survival according to GEMIN6 expression and other prognostic factors.

Characteristics	Total (*n*)	Univariate analysis		Multivariate analysis	
		Hazard ratio (95% CI)	*p* value	Hazard ratio (95% CI)	*p* value
T stage (T3-4 vs. T1-2)	523	2.317 (1.591-3.375)	*<0.001*	1.697 (1.029-2.799)	*0.038*
N stage (N1-3 vs. N0)	510	2.601 (1.944-3.480)	*<0.001*	1.966 (1.332-2.901)	*<0.001*
Pathologic stage (stage III-IV vs. stage I-II)	518	2.664 (1.960-3.621)	*<0.001*	1.312 (0.828-2.080)	0.247
Primary therapy outcome (PR & SD & PD vs. CR)	439	2.690 (1.918-3.771)	*<0.001*	2.308 (1.620-3.286)	*<0.001*
Gender (male vs. female)	526	1.070 (0.803-1.426)	0.642		
Race (White vs. Asian & Black or African American)	468	1.475 (0.902-2.411)	0.121		
Age (>65 vs. ≤65)	516	1.223 (0.916-1.635)	0.172		
Smoker (yes vs. no)	512	0.894 (0.592-1.348)	0.591		
Number pack years smoked (≥40 vs. <40)	363	1.073 (0.753-1.528)	0.697		
GEMIN6 (high vs. low)	526	1.529 (1.136-2.058)	*0.005*	1.491 (1.063-2.092)	*0.021*

## Data Availability

All data are available from the corresponding authors under reasonable conditions.
